# Fixed-Diamond Abrasive Wire-Saw Cutting Force Modeling Based on Changes in Contact Arc Lengths

**DOI:** 10.3390/mi14061275

**Published:** 2023-06-20

**Authors:** Lie Liang, Shujuan Li, Kehao Lan, Jiabin Wang, Ruijiang Yu

**Affiliations:** School of Mechanical and Precision Instrument Engineering, Xi’an University of Technology, Xi’an 710048, China

**Keywords:** contact arc length, cutting force, random distribution of abrasive particles, wire bow angle

## Abstract

Monocrystalline silicon is widely used in the semiconductor market, but its hard and brittle physical properties make processing difficult. Fixed-diamond abrasive wire-saw (FAW) cutting is currently the most commonly used cutting method for hard and brittle materials due to advantages such as narrow cutting seams, low pollution, low cutting force and simple cutting process. During the process of cutting a wafer, the contact between the part and the wire is curved, and the arc length changes during the cutting process. This paper establishes a model of contact arc length by analyzing the cutting system. At the same time, a model of the random distribution of abrasive particles is established to solve the cutting force during the cutting process, using iterative algorithms to calculate cutting forces and chip surface saw marks. The error between the experiment and simulation of the average cutting force in the stable stage is less than 6%, and the errors with respect to the central angle and curvature of the saw arc on the wafer surface are less than 5% between the experiment and simulation. The relationship between the bow angle, contact arc length and cutting parameters is studied using simulations. The results show that the variation trend of the bow angle and contact arc length is consistent, increasing with an increase in the part feed rate and decreasing with an increase in the wire velocity.

## 1. Introduction

As the first-generation semiconductor material, monocrystalline silicon accounts for 90% of the semiconductor market [1] due to its excellent thermal stability, low linear coefficient of thermal expansion, high melting temperature, and ease of preparing large-sized crystal circles [2,3]. Meanwhile, due to the high strength and hardness of monocrystalline silicon, processing technology has always been a hot research topic [4]. The slicing of monocrystalline silicon is the primary process of processing [5], accounting for about 30% of the total cost of the entire process [6,7]. Wire cutting technology has become the commonly used cutting method for hard and brittle materials due to its advantages, such as high processing yield and small incision loss [8,9,10].

With respect to wire cutting technology, there are two types of technology based on the abrasive particles of different states: loose abrasive particles and fixed abrasive particles [11]. Compared with loose abrasive wire cutting technology, fixed abrasive wire cutting has the advantages of low pollution and high efficiency [12]. FAW has become the main cutting method for cutting hard and brittle materials [13,14,15]. The quality of the wafer is the evaluation standard of processing technology. Research has shown that cutting force is closely related to wafer break and wafer quality during the cutting process [16,17]. Cutting force has become the most important process variable in the machining process.

The main factors that affect cutting force in the FAW process include the following: part feed rate, wire velocity and wire pretension. Li et al. [2] found that the cutting force was linearly related to the part feed rate, contact length and wire radius and inversely proportional to the wire velocity. The study of contact arc length here focuses on the contact between the cylindrical part and the wire. Wang et al. [16] pointed out that the cutting forces are key factors affecting the surface quality and sub-surface quality of wafers. Therefore, a cutting force prediction method considering cutting process parameters was established, which combines the brittle fracture removal and ductile removal of single abrasives. Wang et al. [18] proposed a theoretical force model for single diamond abrasive scratching the sigle crystal SiC at any deflection angle, and they established a simplified model for abrasive forces. Wang Yan et al. [19] established a force model for a single abrasive particle based on a constant cutting depth. These studies start from an analysis of the force acting on a single abrasive particle and then study the sawing force during the cutting process. Huang et al. [20] conducted an experimental study on the cutting force in the cutting process of sapphires using a fixed diamond wire saw. The results showed that the crystal structure of sapphire, the reciprocating speed of the wire saw and the part feed rate had an impact on the tangential cutting force. The cutting force of the wire saw increased with a decrease in wire velocity and an increase in feed speed. The tangential sawing force had a good linear relationship with the material removal rate. Some researchers used simulation software to analyze the cutting process. Ge et al. [21] conducted finite element analysis on the cutting stress in fixed abrasive wire-saw machining and analyzed the changes in cutting force during the cutting process. Tang et al. [22] used the finite element method to establish a cutting force simulation model for cutting hard and brittle materials with multiple abrasive-particle wire saws. The simulation results showed that as the wire velocity decreased or the part feed rate increased, the cutting depth of single abrasives during the sawing process increased, leading to an increase in cutting force. Wallburg Florian et al. [23] analyzed and assumed that there was a relationship between the force and the material removal rate. The finite element model was used to calculate the material removal coefficient. Ningchang Wang et al. [24] reported that diamond wire saws cut sapphire in different directions, resulting in different surface qualities. The surface quality is better on the side inflicted with a lower cutting force. Zhishu Lin et al. [25] studied the influencing factors of the wire bow angle and found that the maximum wire bow deflection had an approximately linear relationship with the force ratio and that the force ratio was a key factor influencing the maximum wire bow deflection. Jian Qiu et al. [26] verified, by conducting experiments, that the wire bow shape is related to the cutting direction and cutting force. Liedke and Kuna [27] considered the balance between material removal and cutting force and established a macroscopic mechanical model to describe the deformation of the wire and cutting force during steady-state cutting processes using different process parameters. Zhiyuan Lai et al. [28] found that wire velocity and part feed rate had a significant impact on the value of normal forces. The contact state between the part and the wire, cutting force and material removal are three mutually influencing factors, and the contact state determines the cutting force and its distribution.

References [23,28] point out that many studies on micromechanisms have resulted in fixed-diamond wire cutting research, but a deeper understanding of the macroscopic interaction between the part and the wire saw is lacking. Most studies on macroscopic forces use experimental summary and fitting methods or simulation software to analyze the cutting process. During the cutting process, the cutting force, wire bow angle and contact length between the part and the wire all vary over time and are interrelated. The existing research on cutting force mainly focuses on the average force in the stable stage, while the research on bow angle and contact length is mainly based on experimental measurements. This paper analyzes the process of fixed abrasive diamond wire cutting square monocrystalline silicon and establishes a mathematical model of the contact arc length based on geometric relationships. Based on the relationship between the force on a single abrasive particle and the depth of indentation and considering the random distribution of abrasive particles, an iterative algorithm is used to perform analytical calculations on the entire cutting process. This algorithm can predict the trend of cutting force changes during the complete cutting process and predict the surface morphology of the wafer.

## 2. Cutting Force Model

### 2.1. Modeling of the Contact Arc Length

**Figure 1:**
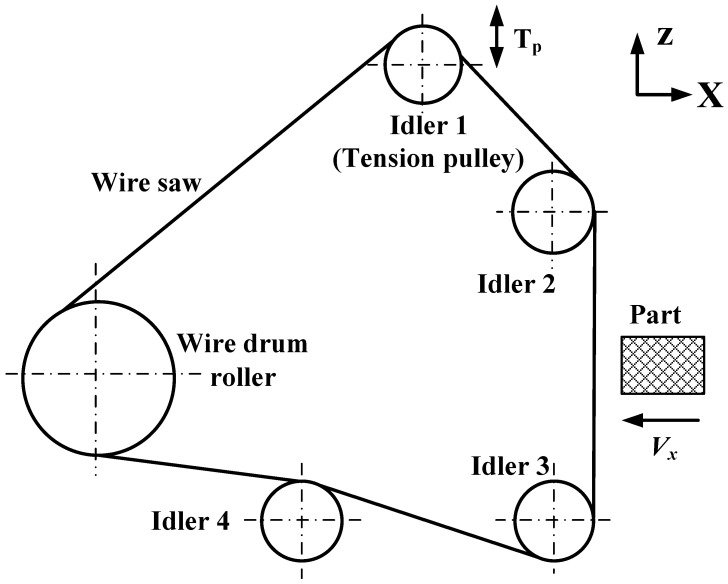
Schematic diagram of monocrystalline silicon cutting.

The FAW system consists of a wire drum roller that drives the motion of the wire and idlers that support the wire, as shown in Figure 1. Idler 1 is the adjustment wheel that tightens the wire after winding, while idler 2 and idler 3 make the wire perpendicular to the direction of the part’s movement. After winding the wire around the drum and idlers, the tension pulley moves upwards to increase the tension of the saw wire. T_p_ is the initial tension force provided by the tension pulley for the wire. During the cutting process, there are directional switches on both sides of the drum. When moving in one direction and touching a switch on one side, the drum stops moving in the opposite direction until it touches the switch on the other side, repeating the movement and forming a reciprocating motion that drives the saw wire to move clockwise and counterclockwise.

**Figure 2:**
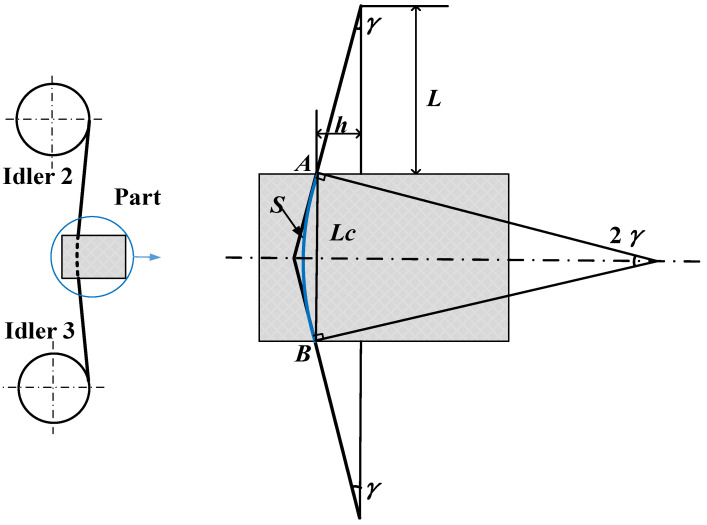
Schematic diagram of the contact arc length between the wire saw and part.

Wire cutting belongs to flexible machining, and the wire bends during processing, resulting in bow angle *γ*, as shown in Figure 2. When cutting a square part, the contact length between the wire and the part is often considered as the height of part *L_c_*. In fact, the shape of the part in contact with the line is an arc rather than a straight line, exhibiting a length of *S*. Assuming that the part is centered and installed between idler 2 and idler 3, the contact arc length is geometrically derived as Equation (1). The contact arc length is related to wire bow angle *γ*. According to geometric relationships, bow angle *γ* is related to *L* and *h*, as shown in Equation (2). Here, *L* is the distance between the upper end of the part and idler 2 after the part is installed, and this value remains unchanged during the cutting process. The reason for the change in bow angle is the change in *h* during the cutting process. The formation of *h* is due to the insufficient removal material ability of the wire saw in the early cutting stage, and the continuous feeding of the part causes material accumulation. *h* remains unchanged only until the amount of material removed by the wire saw is equal to the amount that should be removed by the part feed. The analysis shows that contact arc length *S* is related to *h*.

(1) $$S=\frac{L_{c}}{\sin\left(\gamma \right)}\gamma$$

(2) $$\gamma =\tan^{-1}\left(\frac{h}{L}\right)$$

Here, *S* is the contact arc length, *γ* is the wire bow, *L* is the distance between the centers of idlers 2 and the upper end of the part, *L_c_* is the thickness of the part, and *h* is the bending distance of the wire in Figure 2.

When wire bow angle *γ* is relatively small, *γ*/sin(*γ*) can be approximately equal to 1, which means that contact arc length *S* is approximately equal to the thickness of part *L_c_*. In fact, the wire bow angle changes throughout the entire cutting process. At the initial moment, the bow angle is close to 0, and the contact arc length is close to the thickness of the part. In a later cutting stage, the contact arc length changes with a change in the wire bow’s angle.

### 2.2. Modeling of Abrasive Particle Distribution

**Figure 3:**
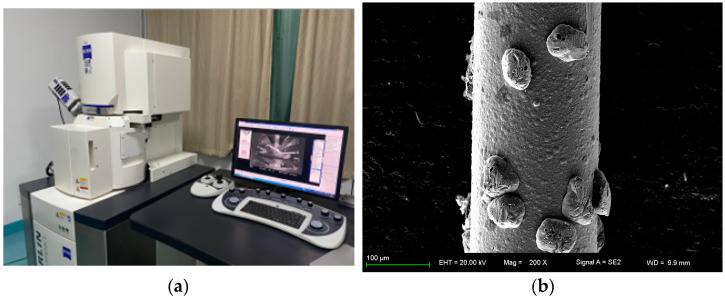
SEM and the photo of the wire saw. (**a**) The Merlin Compact SEM. (**b**) SEM photo of the wire saw.

The Merlin Compact SEM is used, as shown in Figure 3a, and a photo of the wire saw’s surface is taken, as shown in Figure 3b. The extra high tension (EHT) is 20.00 kV, the working distance is 9.9 mm, the magnification (Mag) is 200 times, and the detector is SE2. The abrasive particles on the surface of the wire saw are randomly distributed. The coordinate values of abrasive particles can be given using a random function in MATLAB, and the boundary of abrasive particle coordinates and the number of abrasive particles involved in processing should be determined first.

**Figure 4:**
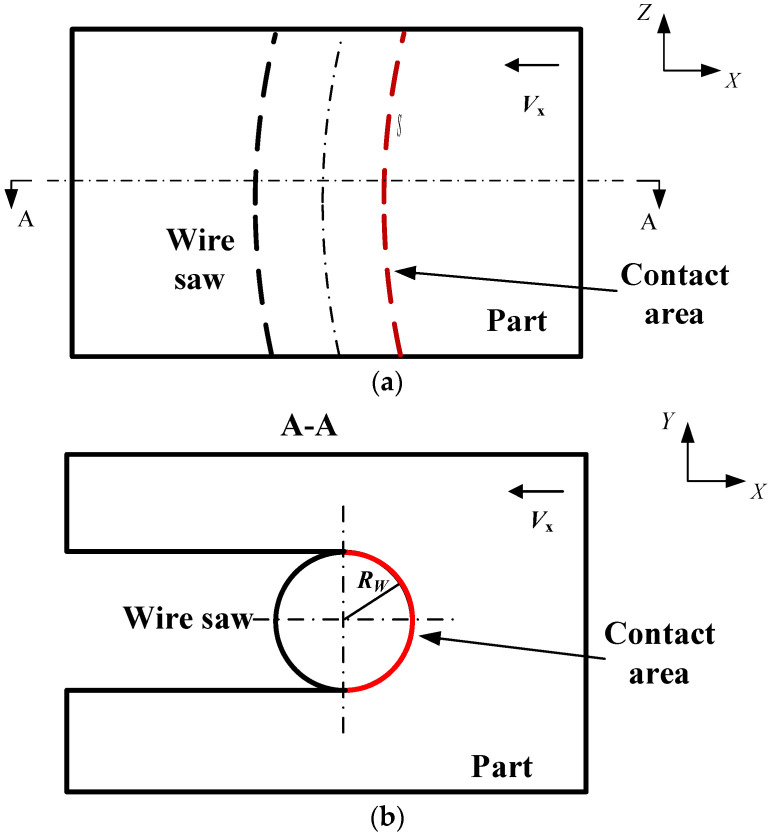
Schematic diagram of the contact range between the part and the wire saw. (**a**) Front saw. (**b**) A-A section view.

The boundary of the abrasive coordinate is the contact range between the wire saw and the part. The contact length is *S*, as shown in Figure 4a and as observed in Section 2.1. The width of contact is half the circumference of the wire saw, as shown in Figure 4b. The coordinate range of abrasive particles is set to [0, *S*] and [0, *πR_w_*]; here, *R_w_* is the radius of the wire saw.

**Figure 5:**
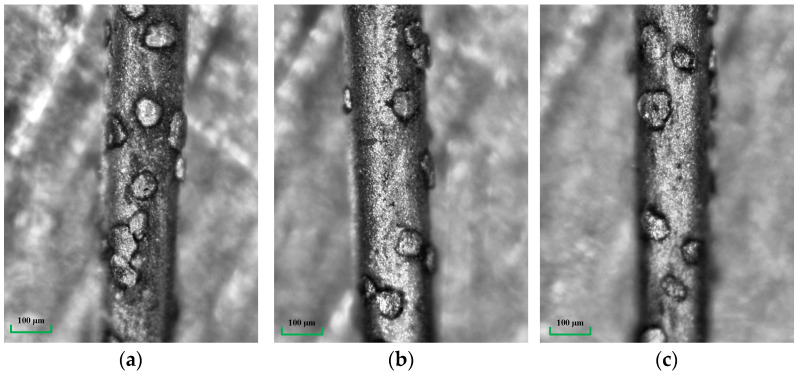
Surface topography of different parts of wire saw. (**a**) Stacking distribution, (**b**) spare distribution, and (**c**) normal distribution.

The wire matrix is nickel-plated and impregnated with JR2-type diamond abrasives. The radius of saw wire *R_w_* is 0.1 mm. The density of abrasive particles is the number of abrasive particles per unit area. The surface morphology of different parts of the wire saw is collected, multiple possible distribution features in Figure 5 are considered, and 100 of them are selected for statistical analysis. The results indicate that the distribution density of abrasive particles on the wire surface is 34 abrasive particles per square millimeter.

The height of abrasive particles obeys the normal distribution, and the probability density function is described in Equation (3) [22]. An excessive abrasive size may lead to rapid wear, while abrasive sizes that are too small do not work in the cutting process, so not all abrasive particles participate in the cutting process. Assuming that the height of the abrasive particles involved in processing is within one standard deviation range in Equation (3), the proportion of abrasive particles involved in cutting processing is approximately *k* = 68%. The contact area between the part and the wire saw is described in Equation (4), and the number of abrasive particles involved in processing within the contact range is calculated using Equation (5):

(3) $$f\left(d\right)=\frac{1}{\sigma_{1}\sqrt{2\pi }}\exp\left[-\frac{1}{2}\left(\frac{d-\mu_{1}}{\sigma_{1}}\right)\right]$$

(4) $$A=\pi R_{w}S$$

(5) $$N=kAC=k\pi R_{w}SC$$

where *d* is the particle size of the abrasive particles, *σ*_1_ is the standard deviation of the normal distribution, *μ*_1_ is the average particle size of the abrasive particles, and *C* is the distribution density of abrasive particles on the wire surface.

**Figure 6:**
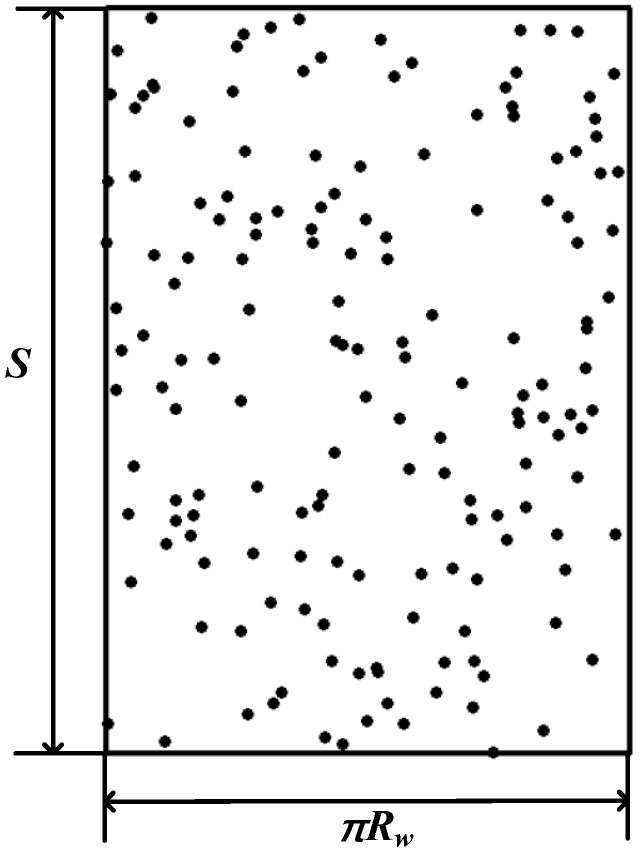
Random distribution result of abrasive particles.

The random function from MATLAB is used to generate the *x*-axis and *y*-axis coordinates of the abrasive particles, where the *x*-axis coordinate range is [0, *πR_w_*] and the *y*-axis coordinate range is [0, *S*]. The established abrasive particle distribution result is shown in Figure 6. If the position of the wire saw in contact with the part changes, the random function is used again to establish the abrasive coordinates in order to keep the abrasive position data updated throughout the calculation process.

### 2.3. Cutting Force Model

**Figure 7:**
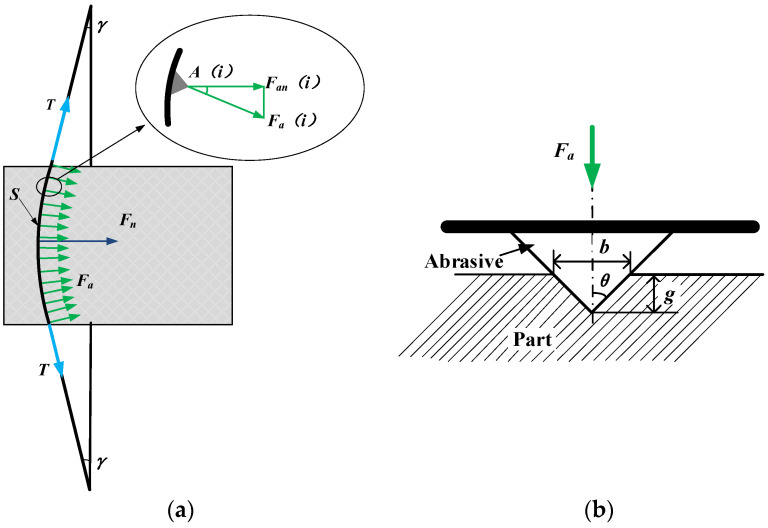
Force analysis of part and abrasive particles. (**a**) Part force analysis. (**b**) Force analysis of abrasive particles.

During the cutting process, the wire saw comes into contact with the part, creating a bow angle and applying pressure to the part. As the part continues to feed, the bow angle increases, and the combined force of the wire saw’s tension in the direction of the part feed increases accordingly. The combined force of the wire saw’s tension in the feed direction of the part is the cutting force of the part, as shown in Figure 7. The relationship between the cutting force and wire tension is shown in Equation (6). After the initial tension setting, the cutting force during the machining process is related to the variation in the bow angle. The change in bow angle can be analyzed in Section 2.1, and it is related to the amount of material removed. According to the analysis in Figure 7, the relationship between cutting force and the force on a single abrasive particle can be represented by Equation (7). According to the research in [14], assuming that the abrasive particles are conical, the relationship between the depth of the abrasive particles pressed into the part and the force is described in Equation (8).

(6) $$F_{n}=2T\sin(\gamma )$$

(7) $$F_{n}=\sum_{i=1}^{N}F_{an}\left(i\right)=\sum_{i=1}^{N}F_{a}\cos\left(\gamma -\frac{2y(i)}{S}\gamma \right)$$

(8) $$F_{a}=\frac{\pi }{2}{\left(g\tan\left(\theta \right)\right)}^{2}H$$

Here, *F_a_* is the positive force on the single abrasive, as shown in Figure 7b, *F_an_* is the component of *F_a_* in the horizontal direction, *g* is the depth of penetration of the abrasive, 2*θ* is the abrasive tip angle, and *H* is the hardness of the part. *F_n_* is the cutting force on the part, *T* is the wire tension, and *y(i)* is the distance from the *i*th abrasive particle to the edge of the contact area between the part and wire.

The volume of material removed by a single abrasive particle is the product of the cross-sectional area of the abrasive particle in the feed direction and the movement distance of the abrasive particle. Here, the movement distance is the contact length between the abrasive particle and the part, which is contact arc length *S*. Therefore, the volume of material removed by a single abrasive particle is

(9) $$Q=g^{2}\tan\left(\theta \right)S$$

The volume of material removed by abrasive particles in contact with the part is

(10) $$U_{w}=Ng^{2}\tan\left(\theta \right)S$$

The material volume corresponding to the part feed length during the corresponding time is

(11) $$U_{p}=2R_{w}S\frac{V_{x}}{V_{s}}$$

Here, *V_x_* is the part feed rate, and *V_s_* is the wire velocity.

Equations (6)–(8) can be used to calculate the depth of the penetration of abrasive *g*, and the material removal amount can be calculated by using Equation (10). The volume of materials that have not been removed during the corresponding time period can be calculated using Equations (10) and (11). The volume of materials that are not removed during the processing is cumulative and requires iterative calculations. By calculating the volume of the unremoved material at each moment, *h* can be obtained, as shown in Figure 1. The wire bow angle is calculated according to Equation (2), and the cutting force at each moment is calculated using Equation (6).

## 3. Simulation and Experiments

### 3.1. Simulation and Experiment System

**Figure 8:**
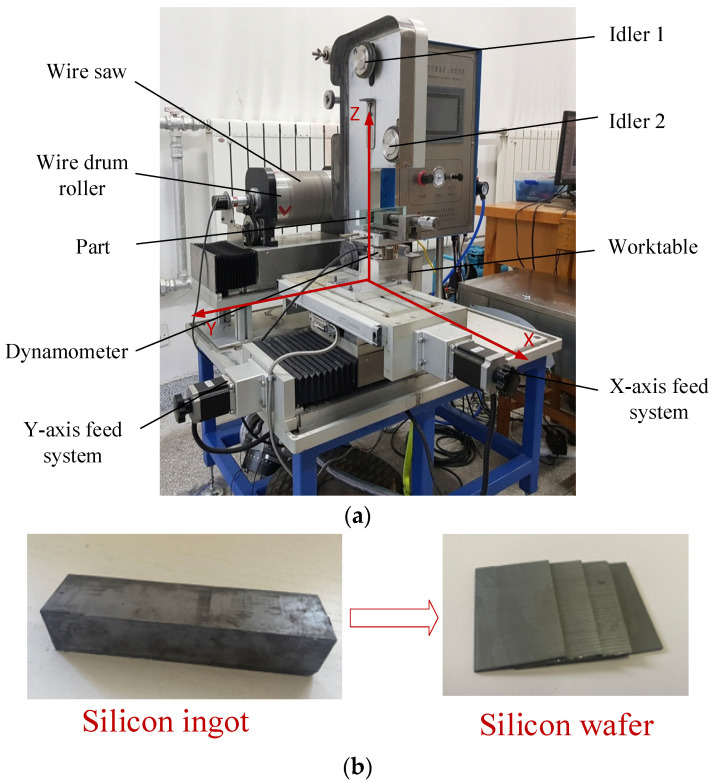
The wire saw machine and schematic diagram of the part before and after cutting. (**a**) The wire saw machine. (**b**) The part before and after cutting.

A WXD170 single-line reciprocating fixed-diamond abrasive wire-saw cutting machine is used for the wire-saw cutting experiment, as shown in Figure 8a, corresponding to the schematic diagram in Figure 1. The part moves in X and Y directions via two linear axes driven by stepper motors. The part comprises a rectangular silicon monocrystal (Si), with a hardness *H* of 13.5 GPa [29]. The silicon ingot is cut into silicon wafers using a wire cutting machine tool, as shown in Figure 8b. The part size is 36 mm × 23 mm × 200 mm, and the cutting surface size is 36 mm × 23 mm. The dynamometer is fixed with the part to measure the forces in the cutting process in real time. The dynamometer is an ATI FT19500, with a resolution of 1/160–1/160–1/80 and a measurement range of 32 N–32 N–100 N in the X–Y–Z direction. According to the research in [30], the cone-apex half angle of the abrasive is taken as 65°. Wire tension *T* is to 15 N. The cutting parameters of part feed rate *V_x_* and wire velocity *V_s_* are changed for cutting experiments.

**Figure 9:**
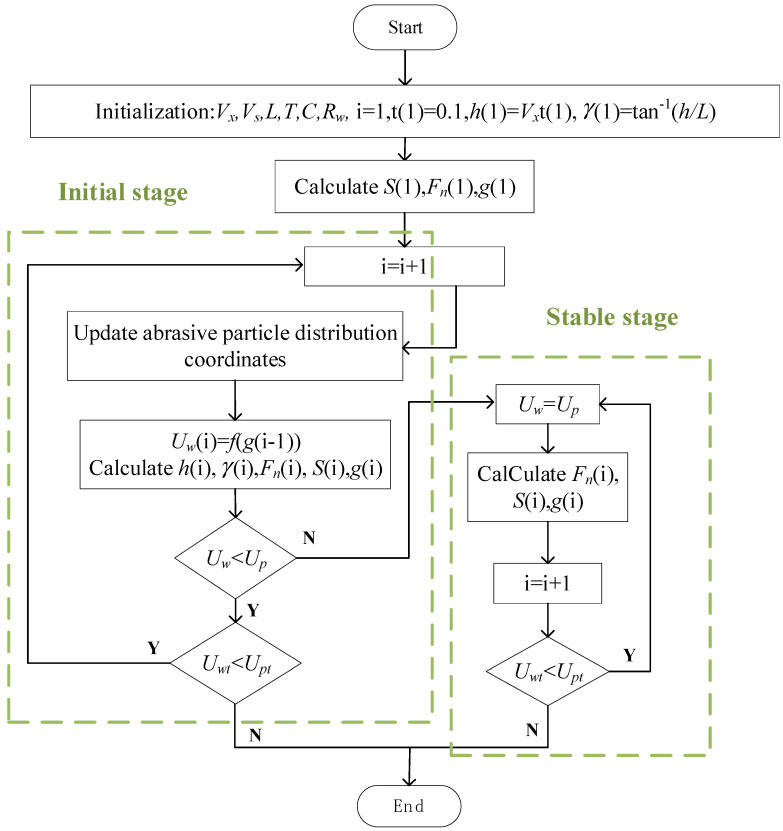
Simulation flowchart.

The simulation uses iterative algorithms, and a flowchart is shown in Figure 9. The simulation is divided into two parts based on the ability of the wire saw to remove materials. In the initial stage, the volume of material removed by the wire saw is smaller than the volume of material that should be removed, corresponding to the part feed rate per unit of time. In the stable stage, both are equal. Here, *U_wt_* is the accumulated value of the actual removed material volume, and *U_pt_* is the total volume of material that needs to be removed to cut off the silicon ingot. When these two are equal, cutting is completed. In the flowchart, there is a situation where the cutting process has not yet entered a stable stage, and cutting is completed. In this case, when the part size is small, cutting is completed during the cutting force increase stage. The update time of abrasive coordinates is the ratio of contact arc length *S* to wire velocity *V_s_*. It is considered that after the abrasive cutting in contact with the part is completed, the coordinates of the abrasive particles are reassigned for calculations.

### 3.2. Results and Discussion

**Figure 10:**
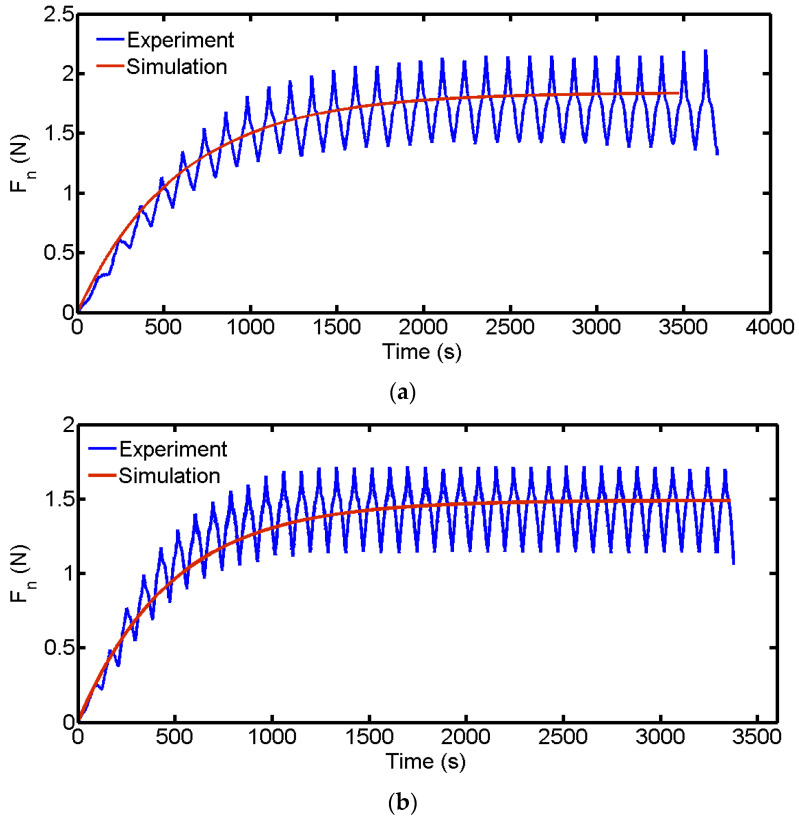
Comparison of the simulation and experimental results of cutting force. (**a**) *V_x_* = 0.75 mm/min and *V_s_* = 1.0 m/s. (**b**) *V_x_* = 0.75 mm/min and *V_s_* = 1.5 m/s.

We compare simulation results with experimental results, as shown in Figure 10. In the experiment, a reciprocating wire saw was used for cutting, so the wire saw reversed in terms of direction. The variation in the cutting force exhibits periodic sawtooth changes, corresponding to commutation. The simulation in this article did not consider the impact of a reversing wire saw. The simulation results of the cutting force do not show periodic changes in the sawtooth’s shape compared to the experiment. However, simulation results are consistent with experimental results regarding the changing trend of cutting forces throughout the entire cutting process. During the initial processing stage, due to the limited removal capacity of the wire saw, the accumulation of unremoved materials leads to an increase in cutting force. When the material removal ability of the wire saw matches the feed ability of the part, the average cutting force remains stable. The simulation results show that the cutting force is also divided into two stages: the rising stage and the stable stage. The usability of the model established in this article is demonstrated by carrying out a comparison between experiments and simulations.

In Figure 10, the variation trend of the simulated cutting force is consistent with the experimental cutting force. The initial cutting force increases and gradually reaches a stable stage. To verify the usability of the model, the experimental and simulated cutting forces under different parameters are compared. There are two stages of cutting forces in the cutting process: the rising stage and the stable stage. The cutting force in the rising stage is not easy to quantify, so the average of the stable stage cutting force is calculated and compared with the simulated stable stage cutting force, as shown in Table 1. The simulated cutting force in groups 1–4 in Table 1 is greater than the experimental cutting force, while group 5 is different. In the experiment, a wire saw cuts many wafers. According to the research in [31], the wear state of abrasive particles can affect the cutting force in the experiment, and abrasive wear is not considered in the simulation. In the early stage of abrasive wear, there is a small change in cutting force, while in the stable wear stage, cutting force decreases; moreover, in the later stage of wear, there is a significant change in cutting force. If a new wire is used for cutting in the experiment, the cutting force may be greater than the simulated cutting force, and the cutting force in the middle or later stages of abrasives wear is often smaller than the simulated cutting force. From Table 1, it can be observed that cutting force decreases with an increase in wire saw velocity and increases with an increase in part feed rate. Both results of the simulation and experiment follow this pattern. This is because of the following reasons: Wire velocity increases, the amount of material removed increases, the accumulation of unremoved material lowers, and the wire’s bow angle during the cutting process is small; thus, the component of wire tension in the direction of cutting force is small, and the cutting force is reduced. On the contrary, as the part feed rate increases, the bow angle increases, resulting in an increase in cutting force. Additionally, the error between simulations and experimental cutting force is less than 6%, which verifies the reliability of the model. This model can effectively predict the cutting force generated during the wire-saw cutting process.

**Figure 11:**
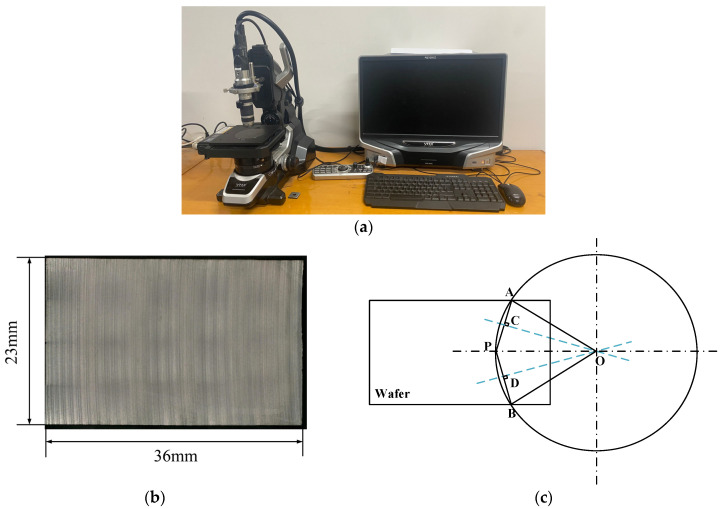
Measurement principle of the wafer’s surface and saw marks after experimental cutting. (**a**) Keyence VHX-5000 ultra depth-of-field 3D microscope (**b**) The surface of the wafer after cutting. (**c**) Schematic diagram of the central angle and radius measurement of the saw mark arc on the wafer surface.

Keyence VHX-5000 ultra depth-of-field 3D microscope (Figure 11a) was used to take photos of the wafer’s surface. The result is shown in Figure 11b, and the distribution of saw marks on the wafer surface can be clearly observed. The measurement of the radius and central angle of the saw trace arc is carried out to find three points of saw traces on the wafer photo. Then, the circle corresponding to the saw trace can be drawn according to the principle of finding the center of three points on the arc. The schematic diagram is shown in Figure 11c. A and B are the intersection points of the saw marks and the edge of the wafer, and P is the midpoint of the saw marks. According to the method of finding the center of the circle using three points, draw the perpendicular line CO passing through the midpoint of the line AP and perpendicular line DO passing through the midpoint of the line BP, respectively. The intersection point O is the center of the circle, OP is the radius, and angle ∠AOB is the central angle corresponding to the wire saw’s trace arc.

**Figure 12:**
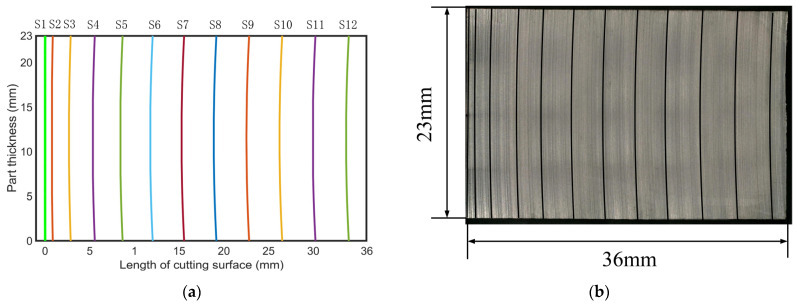
Comparison of wafer surface saw marks between experiments and simulations. (**a**) Simulated wafer surface saw marks. (**b**) Experimental wafer surface saw marks.

During the calculation process of the cutting force at each moment, the contact arc length and bow angle at each moment can be obtained. Figure 12a shows the simulation results of the contact arc length for every 5 min of cutting time. We compare the simulation results with the corresponding saw marks on the surface of the experimental wafer in Figure 12b, as shown in Table 2. The first arc length is at coordinates (0, 0), and this position is close to a straight line in the simulation. The initial curve is treated as a straight line in the process of describing the saw mark in the experiment, so there is no value for the central angle and the curvature of curve 1 in Table 2. Compared with curve 2 to curve 12, the error of the central angle is less than 2.5%, and the curvature error is less than 5%, which shows the reliability of the model in this paper for predicting saw marks on the wafer surface.

**Figure 13:**
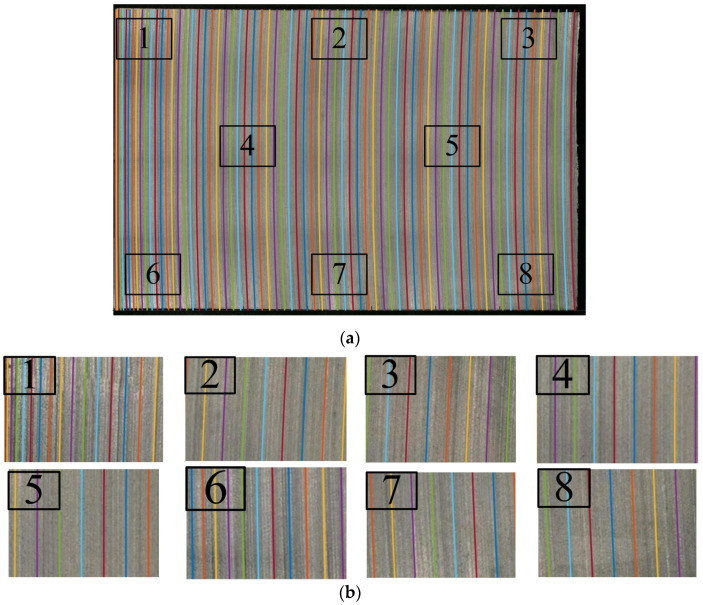
Simulation of saw marks on wafer surface every 50 s. (**a**) Overlay of experimental and simulation saw mark results. (**b**) Partially enlarged view.

The saw marks on the surface of the chip are filled throughout the entire surface. By reducing the simulation’s output time interval to 50 s, the number of simulated saw marks increases and overlaps with the experimental wafer surface, as shown in Figure 13a. Eight different positions on the surface are selected for comparison, as shown in Figure 13b. It can be observed that the simulated saw marks have a good overlap with the chip’s surface. The simulated saw marks are almost parallel to the adjacent saw marks on the experimental wafer. The spacing between saw marks in Figure 13a is not equal. In particular, at the initial moment of cutting, the spacing between saw marks 1 and 6 in Figure 13b is relatively dense. In a later stage of cutting, the spacing between saw marks 3 and 8 in Figure 13b is relatively loose. This also indicates that the material removal ability of wire saws varies throughout the entire process. At the initial stage, due to the weak removal ability of the wire saw, the wire saw’s movement is slow, and the saw marks are dense. In the later stage, the wire saw has strong removal material ability, the wire’s movement is fast, and saw marks are loose.

**Figure 14:**
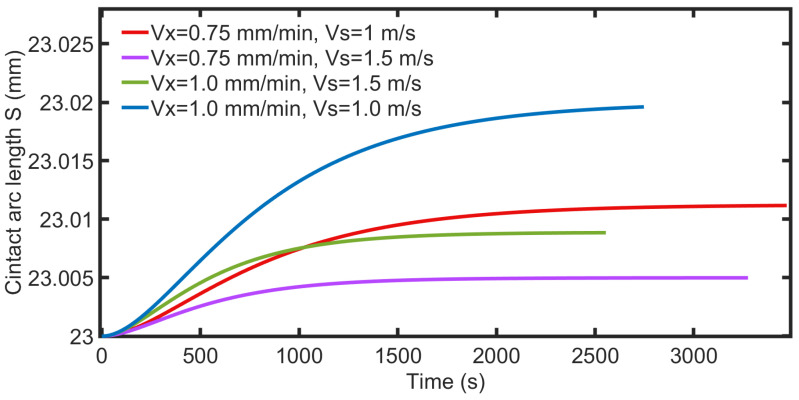
Contact arc length with different processing parameters.

**Figure 15:**
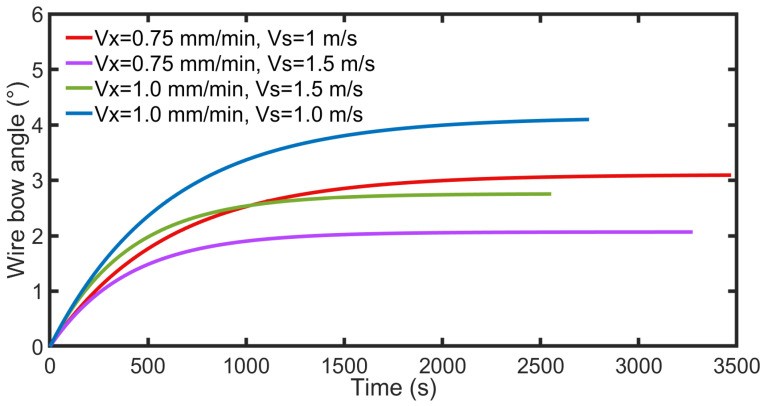
Wire bow angle with different processing parameters.

Furthermore, the simulation results are studied. Figure 14 shows the variation in the arc length during the simulated cutting process. The height of the cutting part is 23 mm, and the variation in the arc length varies at the micrometer level under different parameters. As the wire saw’s velocity increases, the variation in arc length decreases; in contrast, as the part’s feed rate increases, the variation in arc length increases. Figure 15 shows the bow angle under different parameters. Compared with Figure 14, the trend of the changes in bow angle and contact arc length is the same. When the height of the part is determined, the length of the contact arc is related to the angle of the wire bow, as determined by Equation (1). During the cutting process, wire velocity increases, resulting in an increase in the number of abrasive particles in contact with the part per unit time and an increase in the volume of removed material, resulting in a decrease in the bow angle. The increase in part feed rate leads to an increase in the accumulated amount of unremoved material, resulting in an increase in the bow angle. The simulation results show that an increase in both wire velocity and part feed rate can reduce processing times, and an increase in part feed rate leads to a more significant decrease in processing time.

## 4. Conclusions

This paper analyzes the fixed diamond wire cutting system, establishes a random distribution model of abrasive particles on the surface of the wire saw, calculates the change in the contact arc length between the part and the wire saw, and iteratively calculates the cutting force during the cutting process. The errors in cutting force, contact arc length radius and corresponding central angle between the experiment and simulation are less than 6%, which verifies the reliability of the model:

(1) During the wire-saw cutting process of square monocrystalline silicon, the change in cutting force is mainly divided into two stages: the rising stage and the stable stage. The main reason for this phenomenon is the change in the ability of a wire saw to remove materials. In the initial stage, the bow angle is small, the tension component in the feed direction of the part is low, the depth of abrasive particles pressing into the part is shallow, and the volume of material removal is low, resulting in the accumulation of unremoved material. As the accumulation of unremoved materials increases, the wire’s bow angle increases, and the volume of material removal increases. When the removal capacity of the wire saw matches the volume of material that should be removed by the part feed, the cutting system is stable; thus, the cutting force is stable.
(2) When cutting square parts with a fixed-diamond abrasive wire saw, previous studies have believed that the contact part remains unchanged due to its relatively small observed change, which is equivalent to the thickness of the part. By carrying out analyses and calculations in this paper, the contact arc length changes during the cutting process, and the simulation results also confirm this phenomenon.
(3) The change in contact arc length during the cutting process with different parameters is consistent with the change in the wire saw’s bow angle, which decreases with an increase in wire saw velocity and increases with an increase in the part feed rate. During a slicing process, the cutting time is more sensitive to the influence of the part feed rate compared to the influence of wire velocity.

## Figures and Tables

**Table 1:** Simulation and experimental comparison of cutting forces in the stable stage under different parameters.

Number	Part Feed Rate (mm/min)	Wire Velocity (m/s)	Experiment Cutting Force (N)	Simulation Cutting Force (N)	Error (%)
1	0.75	1.0	1.79	1.81	1.1
2	0.75	1.5	1.42	1.47	3.5
3	0.5	1.0	1.17	1.22	4.2
4	0.5	1.25	0.96	1.01	5.2
5	0.5	1.5	0.90	0.85	5.5

**Table 2:** Comparison of the central angle and curvature corresponding to the simulated and experimental saw mark curves on the wafer surface.

Curve	Central Angle			Curvature		
	Simulation (°)	Experiment (°)	Error (%)	Simulation (m^−1^)	Experiment (m^−1^)	Error (%)
S1	0.1036	0	-	0.07859	-	-
S2	2.5240	2.5	0.96	1.9151	1.8785	1.94
S3	3.9832	3.9	2.1	3.0219	2.9263	3.26
S4	4.8633	4.9	0.74	3.6893	3.6770	0.33
S5	5.3945	5.5	1.9	4.0920	4.1083	0.39
S6	5.7152	5.7	0.26	4.3351	4.2308	2.46
S7	5.9090	5.8	1.87	4.4819	4.3033	4.15
S8	6.0260	6.1	1.21	4.5706	4.5750	0.09
S9	6.0967	6.1	0.05	4.6242	4.5750	1.07
S10	6.1395	6.1	0.64	4.6566	4.5750	1.78
S11	6.1653	6.2	0.55	4.6762	4.6395	0.79
S12	6.1809	6.2	0.31	4.6880	4.6395	1.04

## Data Availability

All data generated or analyzed during this study are included in this published article.
